# Hypertrophic Osteoarthropathy

**DOI:** 10.31662/jmaj.2022-0145

**Published:** 2022-11-30

**Authors:** Hiroki Kojima, Aya Oda, Kenichi Shimane

**Affiliations:** 1 Department of Infectious Diseases, Tokyo Metropolitan Bokutoh Hospital, Tokyo, Japan; 2 Department of Rheumatology, Tokyo Metropolitan Bokutoh Hospital, Tokyo, Japan

**Keywords:** hypertrophic osteoarthropathy, hypertrophic pulmonary osteoarthropathy, lung cancer

A 54-year-old man presented with cough and 3-month history of pain in bilateral knees and fingers. Physical examination revealed digital clubbing (Figure 1a and b) and tenderness in the knee joints. Bone scintigraphy revealed areas with technetium 99 m-labeled methylene diphosphonate uptake in the femoral and tibial bones (Figure 1c and d). Thus, he was diagnosed with hypertrophic osteoarthropathy. Chest computed tomography revealed a larger nodule with multiple small nodules in the upper lobe of the right lung (Figure 1e, arrow), as well as right-sided subclavian, hilar, and mediastinal lymphadenopathy. Bronchoscopy was performed, and he was diagnosed with lung adenocarcinoma (T3N3M0). After the initiation of chemoradiation therapy, the pain gradually resolved.

**Figure 1.**
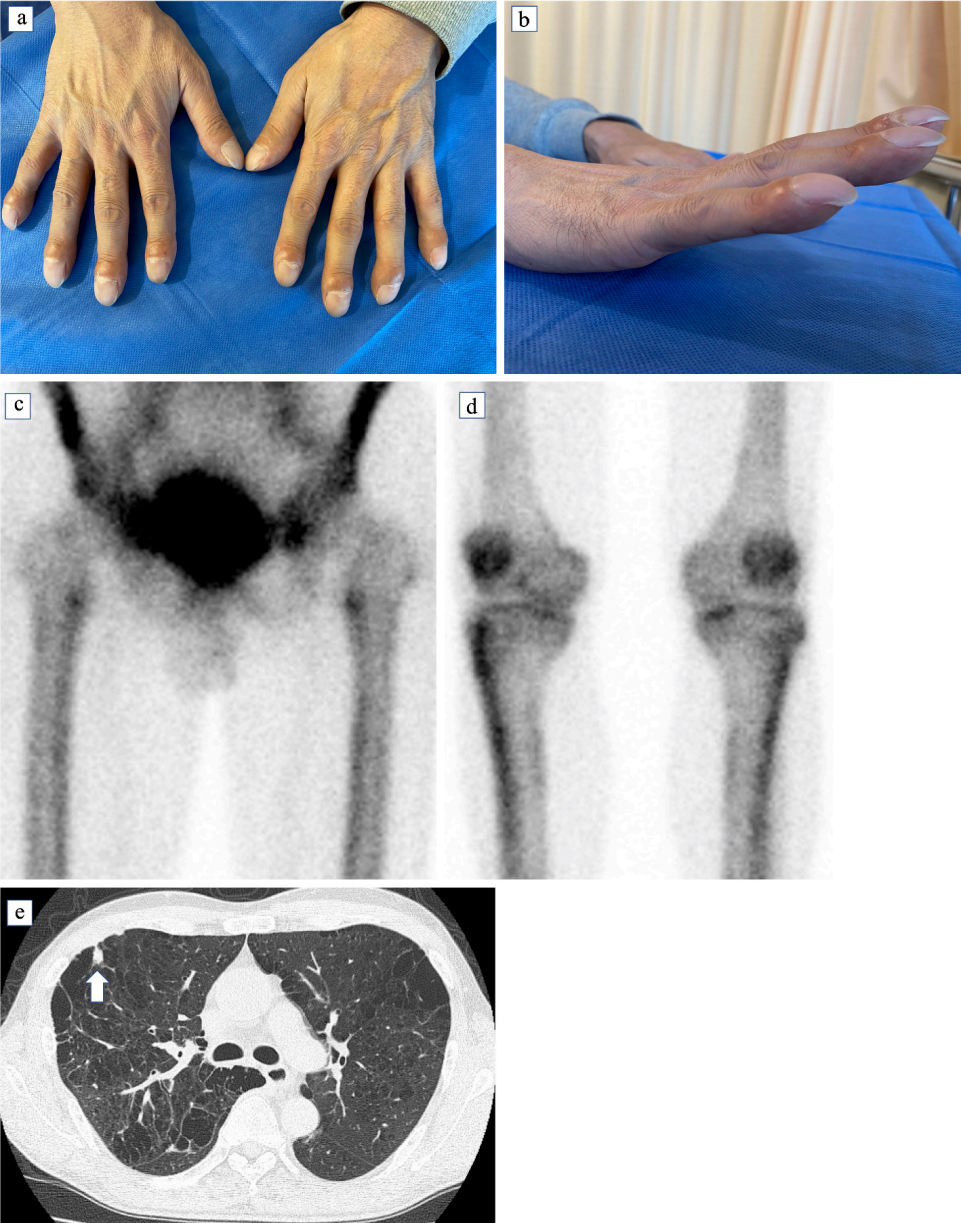
(a), (b): Clubbing of the fingers. (c), (d): Bone scintigraphy revealing areas of technetium 99 m-labeled methylene diphosphonate uptake in the femoral (c) and tibial bones (d). (e): Chest computed tomography showing a nodule in the upper lobe of the right lung.

Hypertrophic osteoarthropathy is characterized by digital clubbing accompanied by arthralgia and periostosis and typically involving the tibia and fibula ^(1)^. 95%-97% of the cases are caused by underlying conditions; the most common being non-small cell lung carcinoma ^(2)^. Screening for underlying conditions, particularly lung cancer, should be performed after hypertrophic osteoarthropathy diagnosis.

## Article Information

### Conflicts of Interest

None

### Author Contributions

HK wrote the manuscript, and AO and KS critically revised the manuscript for the intellectual content. All authors have read and approved the final manuscript.

### Informed Consent

Written informed consent was obtained from the patient.

### Approval by Institutional Review Board (IRB)

Ethical approval was waived by the Ethics Committee of Tokyo Metropolitan Bokutoh Hospital.

## References

[1] Callemeyn J, Van Haecke P, Peetermans WE, et al. Clubbing and hypertrophic osteoarthropathy: insights in diagnosis, pathophysiology, and clinical significance. Acta Clin Belgica Int J Clin Lab Med. 2016;71(3):123-30. 10.1080/17843286.2016.1152672 27104368

[2] Yap FY, Skalski MR, Patel DB, et al. Hypertrophic osteoarthropathy: clinical and imaging features. Radiographics. 2017;37(1):157-75. 27935768 10.1148/rg.2017160052

